# Serological Evidence of Rift Valley Fever Virus Circulation in Domestic Cattle and African Buffalo in Northern Botswana (2010–2011)

**DOI:** 10.3389/fvets.2015.00063

**Published:** 2015-11-25

**Authors:** Ferran Jori, Kathleen A. Alexander, Mokganedi Mokopasetso, Suzanne Munstermann, Keabetswe Moagabo, Janusz T. Paweska

**Affiliations:** ^1^Integrated Animal Risk Management Unit, CIRAD, Campus International de Baillarguet, Montpellier, France; ^2^Department of Animal Science and Production, Botswana College of Agriculture, Gaborone, Botswana; ^3^Department of Fish and Wildlife Conservation, Virginia Tech, Blacksburg, VA, USA; ^4^CARACAL, Center for African Resources: Communities, Animals, and Land Use, Kasane, Botswana; ^5^FAO-ECTAD Regional Office, Gaborone, Botswana; ^6^Botswana Vaccine Institute, Gaborone, Botswana; ^7^World Organization for Animal Health (OIE), Paris, France; ^8^Botswana National Veterinary Laboratory, Sebele, Botswana; ^9^Center for Emerging and Zoonotic Diseases, National Institute of Communicable Diseases of the National Health Laboratory Service, Johannesburg, South Africa; ^10^Faculty of Health Sciences, University of the Witwatersrand, Johannesburg, South Africa

**Keywords:** Rift Valley fever, African buffalo, *Syncerus caffer*, neutralizing antibodies, cattle, serology, one health, Botswana

## Abstract

Rift Valley fever (RVF) is endemic in many countries in Sub-Saharan Africa and is responsible for severe outbreaks in livestock characterized by a sudden onset of abortions and high neonatal mortality. During the last decade, several outbreaks have occurred in Southern Africa, with a very limited number of cases reported in Botswana. To date, published information on the occurrence of RVF in wild and domestic animals from Botswana is very scarce and outdated, despite being critical to national and regional disease control. To address this gap, 863 cattle and 150 buffalo sampled at the interface between livestock areas and the Chobe National Park (CNP) and the Okavango Delta (OD) were screened for the presence of RVF virus (RVFV) neutralizing antibodies. Antibodies were detected in 5.7% (*n* = 863), 95% confidence intervals (CI) (4.3–7.5%) of cattle and 12.7% (*n* = 150), 95% CI (7.8–19.5%) of buffalo samples. The overall prevalence was significantly higher (*p* = 0.0016) for buffalo [12.7%] than for cattle [5.7%]. Equally, when comparing RVF seroprevalence in both wildlife areas for all pooled bovid species, it was significantly higher in CNP than in OD (9.5 vs. 4%, respectively; *p* = 0.0004). Our data provide the first evidence of wide circulation of RVFV in both buffalo and cattle populations in Northern Botswana and highlight the need for further epidemiological and ecological investigations on RVF at the wildlife–livestock–human interface in this region.

## Introduction

Rift Valley fever (RVF) is an emerging zoonotic disease of significant public and animal health concern (1, 2). The most important mosquito vectors of RVF virus (RVFV) are members of the subgenera *Neomelaniconion* (genus *Aedes*) and *Culex* (genus *Culex*) (3). Human infections arise from contact with infected animal blood and tissues or from vector-mediated transmission (4). Human to human transmission has not been reported. The majority of human infections is subclinical or associated with moderate to severe, non-fatal, febrile illness, while some patients can develop a hemorrhagic syndrome and/or ocular and neurological lesions. RVF cases in humans may be clinically indistinguishable from other viral hemorrhagic fevers (VHF), such as Ebola virus disease, Marburg virus disease, Crimean–Congo hemorrhagic fever, and Lassa fever (4, 5). In domestic ruminants, RVF occurs as epizootic waves of abortions and deaths of newborns resulting in significant socioeconomic losses. The difficulty in preventing the disease lies largely in the long interepidemic periods which can be up to 15 years or longer in Eastern and Southern African (4, 6). RVF outbreaks have been reported over much of the African continent, Arabian Peninsula, and were most recently detected in countries from the Indian Ocean (2, 7, 8). Data on RVFV epidemiology, including its maintenance and dynamics of transmission in Botswana, are to date, limited.

Rift Valley fever outbreaks have occurred across most of Southern Africa (4) with the most recent significant outbreaks occurring in 2010 in South Africa and Namibia (9, 10), with apparent suspected spread into Southern Botswana during that same period (11). This was the first reported case of RVF infection in animals in the country. However, serological evidence of RVFV exposure in humans had been identified in the early 1960s in the Chobe District (12). In addition in 1986, 27% of a sample of wildlife hunters residing in Chobe National Park (CNP) (*n* = 52) and 3% of a small sample of the population collected across villages in the same district (*n* = 77) were found seropositive to RVFV. During this survey, a limited number of domestic and wild animals were sampled and tested negative with the indirect immunofluorescence assay, but evidence of RVFV exposure in animals could not be found (13). Given the significance of RVF to both human and animal health, this work focused on evaluating exposure to RVFV among livestock and buffalo populations at the livestock–wildlife interface in two major wildlife areas of Northern Botswana: the Okavango Delta (OD) and the CNP.

## Materials and Methods

### Study Area

The districts of Northern Botswana (Ngamiland East, Ngamiland West, and Chobe Disricts) are characterized by large protected land areas where abundant wildlife and livestock occur in close seasonal association around the only permanent sources of surface water in the region, the OD, and the Chobe River. Botswana has three distinct seasons that strongly influence the movement of wild and domestic animals: the wet season (November–March), the cool dry season (April–July), and the hot dry season (August–October). This cyclical variation of rainfall over the years has strong seasonal effects on the geographic distribution of biomass (livestock and wildlife combined) with the highest density of water-dependent animals concentrated near permanent sources of surface water during the dry season; while during the wet season, animals disperse across the landscape using ephemeral sources of water. These seasonal shifts are most pronounced in and around Chobe District, where the effects of seasonal water availability on vegetation are most dramatic (14).

**Figure 1:**
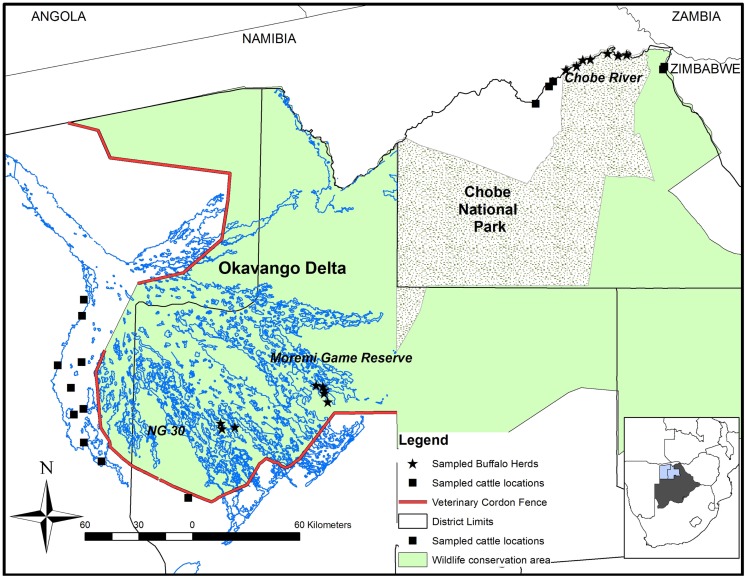
**Map of the study area showing the locations where buffalo and cattle were sampled in Northern Botswana**. The contour of Botswana highlights the Northern Districts of Chobe (right), Ngamiland East (middle), and Ngamiland West (left).

The study was conducted in the OD (Ngamiland East and Ngamiland West Districts) and in the CNP (Chobe District) during October 2010 and October 2011, respectively (Figure 1). Both areas encompass the Botswana component of the Kavango Zambezi Trans Frontier Conservation Area (KAZA TFCA), which connects protected areas from Zambia, Zimbabwe, Namibia, and Angola. Both wildlife areas are within the Botswana Foot and Mouth Disease infected area where buffalo and cattle populations are separated from the primary cattle export regions by buffer zones where movement of animals and related products is controlled. The Chobe River in the north of the CNP constitutes the border between Botswana and Namibia. There is no physical separation between cattle and wildlife in this region. Conversely, the OD, very rich in wildlife, is separated from livestock areas by a double veterinary cordon fence (Figure 1) to prevent contact between buffalo and cattle in the Ngamiland District (15). Buffalo incursions into livestock zones outside the fence occur occasionally, when the fence is damaged by elephants or floods (16). In 2010, buffalo populations were estimated at 31,500 animals in the OD and 7500 in CNP (17).

The total cattle population in Northern Botswana is estimated at 250,000 heads with 2620 cattle occurring in Chobe District and 241,000 cattle in Ngamiland (18). Cattle are mainly indigenous breeds reared in communal grazing areas. As in many other countries in Southern Africa (19), livestock herds sharing the same grazing lands are regularly gathered (approximately twice a month) at crush pens (e.g., holding facilities) used by the Botswana animal health authorities to undertake preventive and control measures for notifiable animal diseases (treatment with acaricides, surveillance and vaccination). The average number of cattle in a crush pen can range between 200 and 2000, the median herd size in northern Botswana being estimated at 35 individuals, interquartile range (19, 64) (16). At the time of the study, Chobe and the two Ngamiland Districts had six and 36 crush pens located, respectively, <15 km from the protected area boundaries, which were used as a focus for cattle sampling.

### Study Design

In buffalo herds, the sampling process was opportunistic and depended on herd detection during aerial capture operations (16). Buffalo sampling was focused along the Chobe River in CNP (*n* = 79) and two additional locations inside the OD: the Moremi Game Reserve (MGR) (*n* = 35) and the NG30 area (*n* = 37). The age of the captured buffalo was estimated from dentition (20). Animals were categorized as young (<1.5 years), subadult (1.5–3 years), or adult (older than 3 years (21). Buffalo densities in these locations were estimated at 1.88 buffalo/km^2^ along the Chobe river, 1.37 buffalo/km^2^ in the MGR, and 3.55 buffalo/km^2^ for the NG30 area (17).

We assumed seroprevalence of RVF to be 5%, the lowest estimated seroprevalence by the virus neutralization test (VNT) in buffalo, in other protected areas in Africa (22). In order to estimate seroprevalence with a 90% confidence level and 5% desired precision, at least 52 animals needed to be sampled per region. We sampled 60 buffalo per wildlife area.

In both districts, cattle crush pens to be sampled were chosen according to their proximity to protected area boundaries and the availability of the National Veterinary Services teams to operate in the area. In this manner, 10 crush pens were selected in Ngamiland District and five in Chobe District. Cattle in this region are regularly vaccinated against FMD, brucellosis, and other diseases but are not vaccinated against RVF.

Data on cattle numbers at crush pens were not available at the time of sampling. From previous fieldwork in the study regions, we estimated cattle numbers at 500 animals per crush pen. Using an estimated seroprevalence of 5% derived from previous studies conducted in sub-Saharan Africa (23, 24), at least 48 animals per crush pen would need to be sampled to estimate seroprevalence with a 90% confidence level and a desired precision of 5%. In the OD interface region, five animals per herd were sampled while 10 animals per herd were sampled in the CNP interface (the first animals available from every herd). With some sample loss due to hemolysis and, at times, lower than expected crush pen numbers in some locations, we obtained a final total sample size of 432 cattle in CNP and 456 cattle in OD.

### Serological Analysis

Sera were tested for the detection of specific anti-RVFV antibodies using VNT (25), the gold standard for detection of RVFV neutralizing antibodies. A serum sample was considered seropositive when it had a titer of ≥log10 1.0, equivalent to a serum dilution ≥1:10.

### Statistical Analysis

Sample sizes were calculated with the use of Epi Tools (http://epitools.ausvet.com.au). Descriptive epidemiological measures were conducted using the software program Epi-Info (CDC, Atlanta, GA, USA) and were reported as percentages of positive animals with 95% Clopper–Pearson binomial confidence intervals (CI). The chi-square test for homogeneity of two populations was used to evaluate the potential influence of age, sex, and capture location on the observed seroprevalence in sampled buffalo, influence of location in sampled cattle, and species and region in the total sample. Stratified analyses were conducted to compare prevalence between species after stratifying by region. Values of *p* < 0.05 were considered significant.

### Ethics

The study was designed and implemented in collaboration with the Food and Agricultural Organization-Emergency Centre for Transboundary Animal Diseases (FAO-ECTAD) Office for Southern Africa. The project was approved by the highest sanitary [Department of Veterinary Services (DVS)] and wildlife management authorities [Department of Wildlife and National Parks (DWNP)] in Botswana. A scientific permit from the DWNP was requested and obtained for sampling buffalo. The sampling of cattle did not require any ethics approval but the project was discussed, approved by DVS, and undertaken with their direct collaboration in the field activities. Permits were obtained from appropriate regulatory authorities for the movement of animal samples outside the foot and mouth disease infected area to targeted laboratories.

## Results

A total of 150 buffalo (79 from CNP and 71 from OD) and 863 cattle (424 from CNP and 439 from OD) samples were finally obtained for analysis and interpretation. Results for the observed prevalence and 95% CI per species and region can be found in Table 1. The overall prevalence was significantly higher (*p* = 0.0016) for buffalo [12.7%, 95% CI (7.8–19.5%)] than for cattle [5.7%, 95% CI (4.3–7.5%)]. When comparing RVF seroprevalence in both wildlife areas for all pooled bovid species, seroprevalence was significantly higher in CNP than in OD (9.5 vs. 4%, respectively; *p* = 0.0004). Stratified analysis of the effect of species after adjusting for region provided that differences in prevalence between species were only marginally significant in CNP (*p* = 0.057) but highly significant in the OD (*p* = 0.005).

**Table 1:** **Occurrence of RVF antibodies per species and region (and age, sex, and capture site in the case of buffalo) based on VN test results expressed in prevalence, ratio and 95% confidence intervals (CI)**.

Species	Variable	Category	Prevalence (%), ratio, and 95% confidence interval	*p* value
Cattle	Location	Chobe National Park	8.5 (36/424) [6.1–11.7]	0.004

		Okavango Delta	2.96 (13/439) [1.65–5.1]	
Buffalo	Location	Chobe National Park	15.38 (12/78) [8.21–25.33]	0.29
	
		Okavango Delta	9.72 (7/72) [4.00–19.01]	
	
	Age	Subadult^a^	9.90 (5/54) [3.08–20.3]	0.07
		Adult^b^	13.48 (12/89) [7.25–22.61]	
	
	Sex	Female	10.1 (9/89) [4.73–18.33]	0.45
		Male	14.3 (8/56) [6.38–26.22]	
	
	Capture site	Chobe River	15.38 (12/78) [8.21–25.33]	
		Moremi Game Reserve	8.57 (3.35) [11.0–26.3]	0.5
		NG 30	10.81 (4/37) [3.0–25.4]	

Overall			6.71 (68/1013) [5.29–8.48]	

*^a^3 years or younger*.

*^b^Older than 3 years*.

### Buffalo

Buffalo were sampled from 15 different herds. The herd size varied widely ranging between bachelor groups of five individuals to megaherds (e.g., temporal aggregation of several subherds potentially reaching more than 1500 individuals). Among the sampled buffalo of known age, 10.5% (*n* = 144) were young animals (1.5 years or younger), 28% (*n* = 144) were subadults (1.5–3 years), and 62% (*n* = 144) were adults (older than 3 years). The median estimated age of the sampled population was 5 years (2–9). Among animals sampled, 59% were female and 37% were male. For a limited number of individuals, age or sex data were not available (seven and five individuals, respectively). These samples were excluded from analyses of sex or age. Neutralizing antibodies against RVFV were detected in 10 out of the 15 herds sampled with values ranging between 6 and 29%. The overall prevalence in buffaloes was [12.7%, 95% CI (7.8–19.1%)] with 84% of those having titers ≥1/40 (*n* = 19). The median age of positive buffaloes was 6.5 years (5–8). Seroprevalence was higher in animals captured in the CNP than OD, but those differences were not statistically significant. Similarly, no significant differences in seroprevalence levels were detected by age class, capture site, or sex (Table 1).

### Cattle

Among the seropositive cattle, 75% (*n* = 49) had titers higher than 1/40. In those animals sampled in OD, five out of 10 crush pens showed detectable neutralizing antibodies at prevalence ranging between 2.1 and 11.6%, while in the CNP, four out of five crush pens detected positive animals, with seroprevalence values ranging between 5.2 and 17.8% (Table 1). Seroprevalence was significantly higher in cattle sampled at the interface with CNP compared to cattle sampled at the OD periphery (*p* = 0.004, 9.5 vs. 3.9%; Table 1).

## Discussion

We report the first large-scale RVFV seroprevalence study in African buffalo and domestic cattle in Botswana. We identified the presence of neutralizing antibodies to RVFV in both species in the absence of reported clinical disease at the time of sampling (October 2010 and 2011). Silent circulation of RVFV has been described elsewhere in sub-Saharan Africa (6) and suggests that in some locations, the virus may circulate for decades, in the absence of reported outbreaks or identification of clinical cases in humans or animals. Levels of antibodies detected in buffalo samples (13%) were considerably higher when compared with those reported in other studies in South Africa [5.8%; *n* = 928 (26), 7.5%; *n* = 1023 (27), and 21/1%; *n* = 66 (28)] and similar to the ones reported during an interepidemic period in Kenya [15.6%; *n* = 237 (22)].

In cattle, seroprevalence levels were significantly lower than those observed in buffalo, but results were difficult to compare because VNT surveys in cattle are scarce in the literature. Using the VNT to evaluate RVF seroprevalence in cattle in Burkina Faso, Boussini et al. (23) reported an overall prevalence of 11.8% (*n* = 40), but results per herd or area were not provided. Similarly, the sample size in our study was insufficient to determine RVFV exposure status at a herd level and, therefore, only regional prevalence values are provided.

The VNT is a highly accurate test with little or no cross-neutralization with other phleboviruses (29), and it is regarded as the gold standard for RVF serology. Nevertheless, it is laborious, expensive, and since it requires live virus, can only be implemented in suitable biocontainment facilities. Neutralizing antibodies detected by the VNT are life long and therefore, seropositive status does not provide insight into exposure period (29). The age of sampled cattle was not recorded in our study, limiting our ability to evaluate how recently the animals might have been exposed to RVFV. In the case of buffalo, our sample was biased towards adult individuals (59.3% of sampled animals) and only five subadult individuals (3 years or younger) were found seropositive. The youngest seropositive animals detected were 1.5 years (*n* = 2), indicating that the most recent exposure to virus likely occurred <2 years before the animals were sampled. In addition, the median age of positive buffalo was quite high and could partially explain the high levels of antibodies observed in our buffalo sample.

Our findings provide evidence that RVFV is actively circulating at the domestic and wild bovid species at the wildlife–livestock interface in the absence of other reported disease outbreaks or clinical cases. The only exception is a recent confirmed case of RVFV infection presenting abortions in two cows from a farm in the Chobe District (30). This case occurred in the same cattle area where our survey was conducted. In the context of our findings, this single case report suggests that clinical disease may be occurring at higher levels than suspected. This seems to be further supported by the fact that nearly one-third of small scale cattle farmers interviewed in Northern Botswana at the time of this study (16) reported the occurrence of abortions in their herds in the preceding year (31.2%, *n* = 135). Although abortions can be induced by a diversity of pathogens, these data suggest that cattle abortions are common, but often underreported or not investigated at all, due to the extensive production systems prevalent in the study area and the absence of systematic reproductive monitoring in cattle among small scale farmers. Similarly, human cases in local clinics may easily go undetected or possibly misdiagnosed with other common infectious diseases presenting with acute febrile symptoms, such as malaria (31).

Our survey results indicate non-negligible prevalence values in both study areas which are separated by 370 km, suggesting that the presence of RVF is likely to be widespread in Northern Botswana. Seroprevalence values were significantly higher in domestic and wild animals from the Chobe District than in those living inside or surrounding the OD. The fact that the sampling effort ended up being more intense in the CNP interface than in the OD interface (sample sizes were similar but populations of buffalo and cattle are significantly higher in the OD) could partially explain the differences in regional prevalence estimates. However, there are also ecological differences that could affect the dynamics of vectors, hosts, and disease presence. Each of these hydrological systems is exposed to different environmental and climatic influences regulating water levels which can affect mosquito hatching and survival in different ways. In the OD, for instance, large water surfaces are available all year round in the inland delta. In the CNP, water availability is localized in a river floodplain system, which can have an impact in the aggregation patterns of domestic and wild hosts around the banks of the Chobe River during the dry season. Our data further suggest that, as shown in other parts of Southern Africa (28), buffalo might be important as a natural host playing a role in the amplification of RVFV in Northern Botswana. Other wildlife species may also be involved and act as amplification hosts (22, 25, 32, 33). However, the full spectrum of wild and domestic host involvement and their contribution to the virus circulation in Northern Botswana is presently unknown.

Weather conditions are known to affect the dynamics and intensity of RVF outbreaks through influence on both vector and host population dynamics and viral transmission. In the 1986 human survey (13), a higher seroprevalence of anti-RVFV antibodies was detected in December after the onset of heavy rains. Equally, the abortion cases reported in 2014 were detected in April most likely resulting from an infection during the rainy season suggesting that the virus could be more active during the first months of the year and could circulate periodically during the rainy season.

Little information is available regarding the spatial and temporal dynamics of RVFV circulation in Southern Africa at the wildlife–livestock–human interface. Buffalo populations in Botswana within the boundaries of the KAZA TFCA are potentially connected, allowing movement of the virus through larger landscapes. Molecular epidemiological investigations will be needed to provide insight into the spatial and temporal dynamics of transmission and persistence for this important animal and public health threat.

## Conclusion

Our results provide evidence that RVFV circulates actively in both wild and domestic bovids in two different areas of Northern Botswana. This raises concern of the potential exposure of RVFV in humans in the region and the need to consider the possible occurrence of RVF infection in acute febrile and/or hemorrhagic conditions. Similarly, clinical cases in domestic animals may be more common than currently reported, raising the need for increased public awareness on the importance of reporting abortions in livestock. Particular attention should be given to the occurrence of such cases after the onset of heavy rains. Further studies are needed to characterize RVF circulating virus in these endemic settings and investigate the temporal and spatial epidemiological dynamics of virus transmission and circulation at the human, domestic, and wild animal compartments. As RVFV spillover to humans can occur through vector transmission as well as direct contact with infected animal blood and tissues, it will be critical to consider vector ecology and the impact of local human practices that might increase the risk of individual exposure such as veterinary investigation of abortions and activities linked with wildlife hunting and the use of bushmeat.

## Conflict of Interest Statement

The authors declare that the research was conducted in the absence of any commercial or financial relationships that could be construed as a potential conflict of interest.
